# Robust rapid-setting antibacterial liquid bandages

**DOI:** 10.1038/s41598-020-71586-7

**Published:** 2020-09-15

**Authors:** Carlos A. P. Bastos, William D. Thom, Beth Reilly, Iris L. Batalha, Maedee L. Burge Rogers, Ian S. McCrone, Nuno Faria, Jonathan J. Powell

**Affiliations:** 1 Department of Veterinary Medicine, University of Cambridge, Madingley Road, Cambridge, CB3 0ES UK; 2 Department of Engineering, Nanoscience Centre, 11 J. J. Thomson Avenue, Cambridge, CB3 0FF UK

**Keywords:** Biomedical materials, Translational research

## Abstract

Bandaging is a steadfast but time-consuming component of wound care with limited technical advancements to date. Bandages must be changed and infection risk managed. Rapid-set liquid bandages are efficient alternatives but lack durability or inherent infection control. We show here that antibacterial zinc (Zn) and copper (Cu) species greatly enhance the barrier properties of the natural, waterproof, bio-adhesive polymer, shellac. The material demonstrated marked antibacterial contact properties and, in ex-vivo studies, effectively locked-in pre-applied therapeutics. When challenged in vivo with the polybacterial bovine wound infection ‘digital dermatitis’, Zn/Cu-shellac adhered rapidly and robustly over pre-applied antibiotic. The bandage self-degraded, appropriately, over 7 days despite extreme conditions (faecal slurry). Treatment was well-tolerated and clinical improvement was observed in animal mobility. This new class of bandage has promise for challenging topical situations in humans and other animals, especially away from controlled, sterile clinical settings where wounds urgently require protection from environmental and bacterial contamination.

## Introduction

Bandaging is an enduring approach to the medical and surgical management of wounds, with a myriad of benefits; including, provision of structural support, prevention of ingress of dirt and infectious microbes from the environment, and the securing of dressings and topical therapeutics (e.g. antibiotics) at afflicted sites^1–3^. The principal drawbacks of conventional bandages are (1) the inconvenient and/or labour-intensive application that is required (2) the requirement for regular changing, to avoid them becoming niduses for infection and (3) the requirement for antimicrobial under-dressings.

Liquid bandage formulations are potentially well placed to redress these limitations, being facile to apply and intrinsically amenable to modification for added functionality or fine-tuning. Ideally, liquid bandage formulations should produce robust protective barriers, comprise safe, biodegradable components, and may even offer additional functionality to promote wound healing^4^. One approach adopted by the more advanced conventional (i.e. solid) bandages is to impart them with antimicrobial properties^2,5^ such that they remain sterile during use and are not harbingers for biofilm formation. Metal ions are attractive for this purpose, being antibiotic-sparing and having broad-spectrum activity^6^. In particular, silver has seen wide-spread topical usage^5,7–9^ and, although potent as a released ion, it is actually inferior to copper at contact killing^10,11^. Silver, if released into the wound, is also potentially problematic to eukaryotic cells^12,13^. In contrast, copper and zinc are physiologically essential trace-minerals and, if leached into the wound significantly, their antibacterial properties could be harnessed by the innate immune system as part of our natural repertoire for fighting infections^14,15^.

In this work, we employed copper, zinc, and shellac—a natural, safe resin used in confectionary and cosmetics^16^—to develop robust, rapid-setting, liquid bandages with inherent antibacterial repellent (contact killing) properties. For proof-of-principle in vivo application and tolerability we considered a worst-case-scenario challenge, namely digital dermatitis, a chronic, contagious, polybacterial infection that afflicts the feet of dairy cows with painful lesions. It impairs mobility, milk production and quality of life^17,18^. Continuous mechanical stress and exposure to bacterial-rich slurry make topical treatment of digital dermatitis extremely challenging^3^. To narrow down the various formulations for in vivo testing their in vitro efficacy was first determined against *Escherichia coli.* In digital dermatitis the causative organisms are typically anaerobes and very difficult to culture. *E. coli*, in contrast, is a standard laboratory model bacterium that is a facultative anaerobe. However, since copper ions and copper surfaces have broad spectrum antibacterial properties^19,20^ we considered that the ‘*E. coli* test’ would be a suitable triage system to answer the question of whether the material’s metal ions were available for bacterial contact killing or not.

## Results

**Figure 1:**
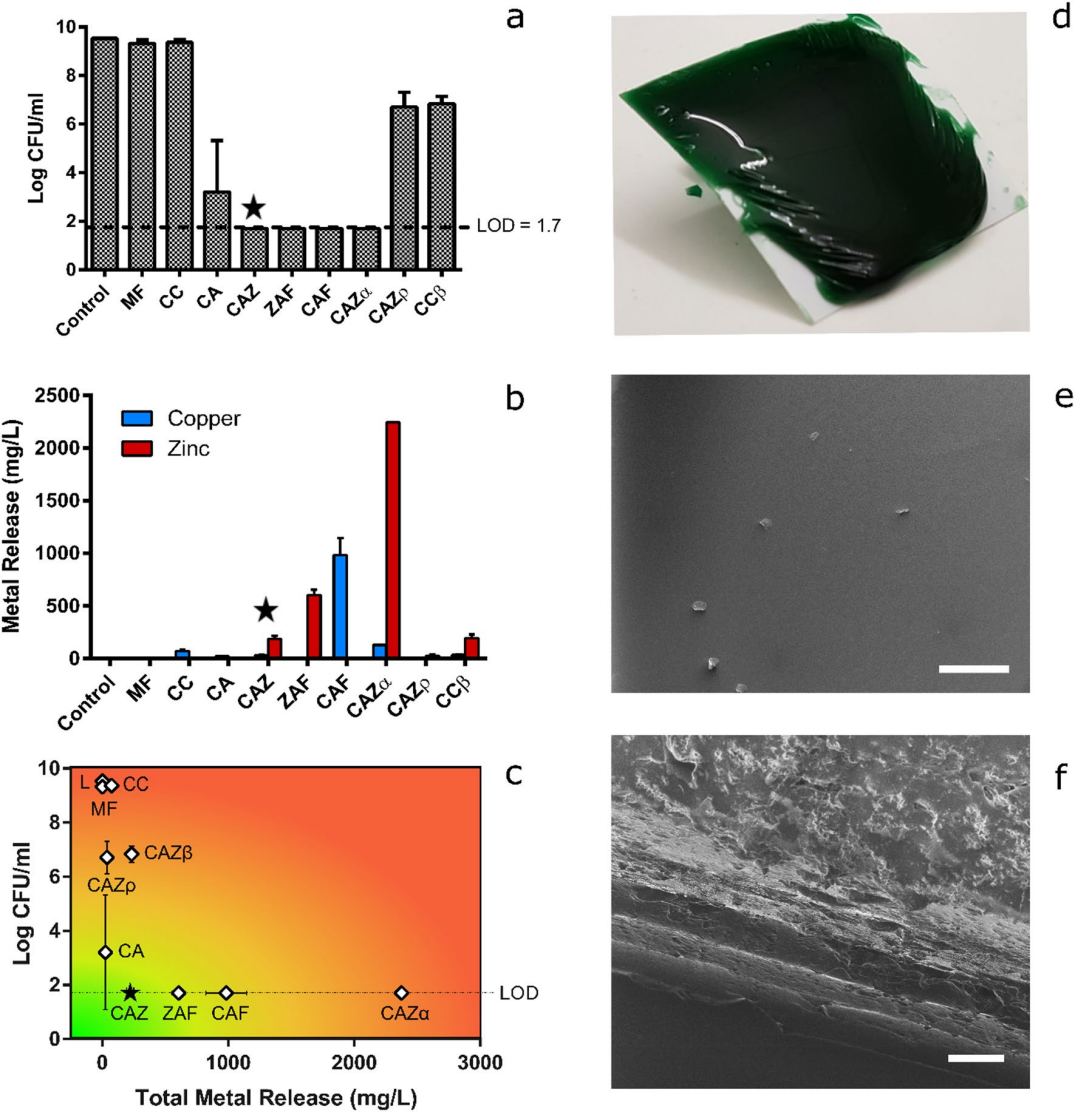
In vitro characterization of liquid bandages. (**a**) *E. coli* concentration (log CFU/mL) after 24 h incubation on a range of barrier surfaces in a dynamic contact killing assay. The dotted line represents the assay’s limit of detection (LOD) of 1.7 log CFU/mL. (**b**) Copper and zinc release from barriers into the culture medium (lysogeny broth) during the contact killing assay as determined by inductively coupled plasma optical emission spectroscopy. (**c**) A combined plot of contact killing ability (log CFU/mL) and total metal release (the sum of copper and zinc), with colour coded outcomes. Ideal material characteristics (antibacterial contact killing with minimal metal release) is indicated by the green region and was best achieved by material CAZ (star symbol). Control (i.e. barrier free) results are denoted by the letter ‘L’. (**d**) Exemplification of barrier formation upon application of CAZ to a polyethylene surface. Helium ion microscopy images of (**e**) surface (scale bar, 200 µm) and (**f**) cross section (scale bar, 50 µm), respectively, of the CAZ barrier. Error bars represent experimental standard deviations**.**

Shellac is highly soluble in ethanol and its solutions readily yield solid adhesive layers upon evaporation: i.e. after being applied to surfaces^16^. In the initial phase of development, we therefore assessed, visually, how well copper- and zinc-based materials mixed with shellac/ethanol solutions. Both simple salts (i.e. copper or zinc chloride) and acetate complexes integrated well into shellac/ethanol solutions. Moreover, in the semi-quantitative retention assay, the presence of these metal species appeared, generally, to *enhance* shellac barrier adhesion on a semi-rigid fabric surface exposed to an aqueous environment (Supplementary Figure S1). Before assessing this formally, we sought to hone in on a lead material based upon antibacterial effectiveness and stability of the antibacterial metals in the barrier. As such, a series of nine rapid-setting liquid materials were produced by varying the metal:shellac:ethanol ratios, the nature of the metal compound, and/or the addition of plasticizers, as described in Materials and Methods and summarised in Table 1. In the contact killing assay against *E. coli*, four materials resulted in undetectable bacterial counts at 24 h (Fig. 1a): these were termed CAZ, ZAF, CAF and CAZα (see Table 1 for material descriptions). Each of these released some metal ions into the bacterial medium over the same time period but, of the four, release was lowest for CAZ, which contained 100 mMol/kg copper acetate and 100 mMol/kg zinc chloride (Fig. 1b). CAZ, therefore, was the preferred option for downstream application to digital dermatitis lesions, due to its ability to contact kill bacteria without substantial leaching of its metal ions into an aqueous environment (Fig. 1c). However, prior to in vivo testing we confirmed that CAZ should be ‘fit for purpose’ through further in vitro tests. Structurally, CAZ formed thick and robust barriers (Fig. 1d). Imaging via helium ion microscopy revealed smooth surfaces of even contrast (Fig. 1e), indicating a homogenous surface layer^21^. However, similar cross-sectional imaging of CAZ films revealed distinct striations (Fig. 1f) consistent with either auto-stratification^22,23^ or significant variation in local topography^24^.

**Table 1:** Identities and components of prepared liquid bandage formulations. All materials contain shellac at 40% (weight by weight) in ethanol solvent.

Material name	Copper	Zinc	Additives
MF	–	–	–
CC	CuCl_2_	–	–
CA	CuAc_2_	–	–
CAF	CuAc_2_	–	FeCl_3_
ZAF	–	ZnAc_2_	FeCl_3_
CAZ	CuAc_2_	ZnCl_2_	–
CAZα	CuAc_2_	ZnCl_2_	ZnO; triethyl citrate
CAZp	CuAc_2_	ZnCl_2_	Polyethylene glycol (~ 400 Da); glycerol
CCβ	CuCl_2_	ZnCl_2_	Benzoic acid

**Figure 2:**
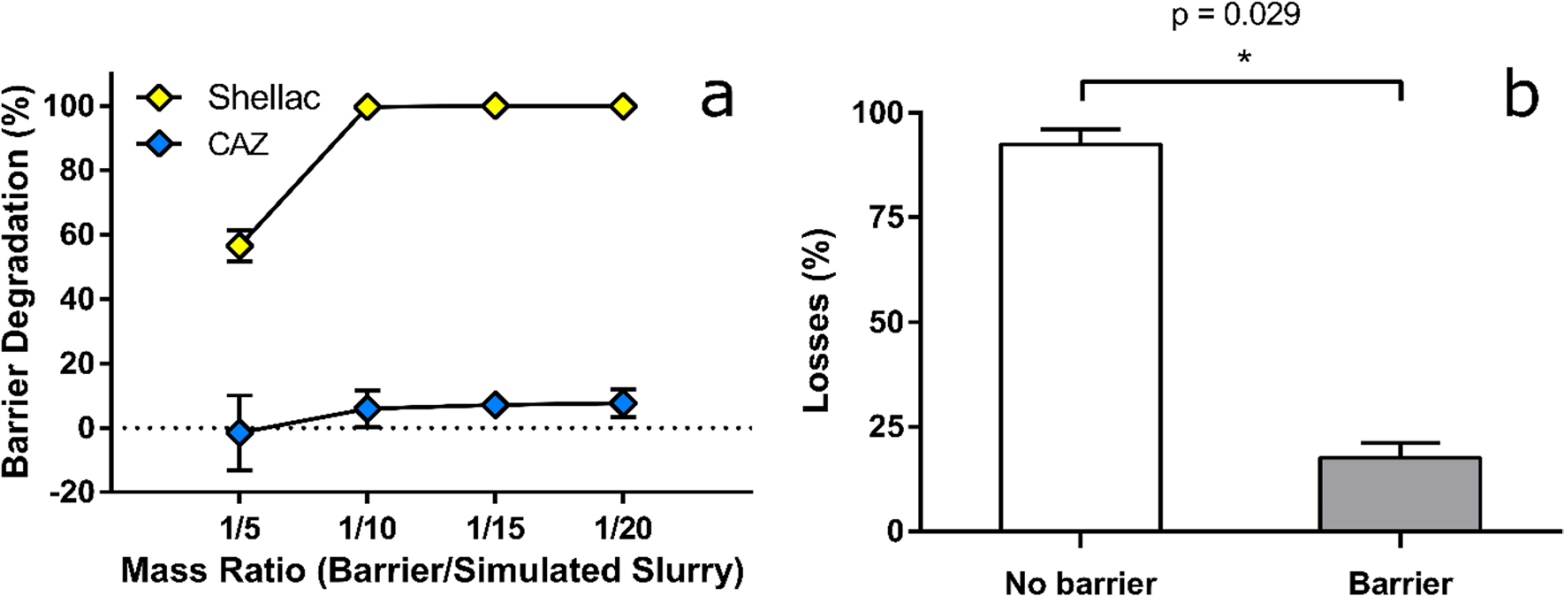
Barrier degradation and antibiotic locking. (**a**) Mass losses of barriers upon exposure to increasing amounts of simulated slurry for dried materials without (shellac) or with (CAZ) the presence of antimicrobial metal ions in their formulation (n = 3). (**b**) Ex vivo losses of antibiotic spray (quantified by loss of its dye, Patent Blue V) in simulated slurry (75 mMol/kg ammonium carbonate; n = 4) in the absence or presence of the CAZ barrier, with statistical analysis via two-tailed Mann–Whitney *U* test. Error bars represent experimental standard deviations.

Importantly, in assays mimicking exposure to alkaline slurry (pH 8.5), as encountered by digital dermatitis lesion-bearing and infected hooves on dairy farms, CAZ barriers were highly resistant to degradation over 24 h (Fig. 2a) regardless of exposure volume (i.e. barrier to slurry ratio). This contrasts with barriers produced by an equivalent metal-free shellac (40% w/w) formulation, which showed only some resistance under the mildest conditions (assay ratio of 1:5) but were fully degraded by greater volumes of slurry-ratios of 1:10 and higher (Fig. 2a). Subsequent ex vivo assays assessed the effectiveness of CAZ barriers at ‘locking in’ pre-applied therapeutics. Typically, digital dermatitis treatment entails repeated applications of antibiotic spray to an affected area until a healing lesion is achieved^25^. Sprays comprise an active antimicrobial (e.g. oxytetracycline or chlortetracycline) and added colorants to enable visualisation of the treatment. Here, leaching of blue dye from a chlortetracycline-based spray was used to demonstrate the ‘lock-in’ effectiveness of CAZ, versus no barrier (antibiotic spray alone; current gold standard), on bovine cadaver legs. Spray was applied between the interdigital cleft and dew claws of each leg. After a brief drying period (60 s), legs were either directly immersed in 1 L of simulated slurry for an hour (non-barrier group), or bandaged with CAZ and, after a 2 min setting period, immersed in 1 L simulated slurry (barrier group). Notably, almost 90% of the antibiotic, quantified via the dye-proxy, was retained with the CAZ barrier, in contrast with less than 15% for the non-barrier group (Fig. 2b).

**Figure 3:**
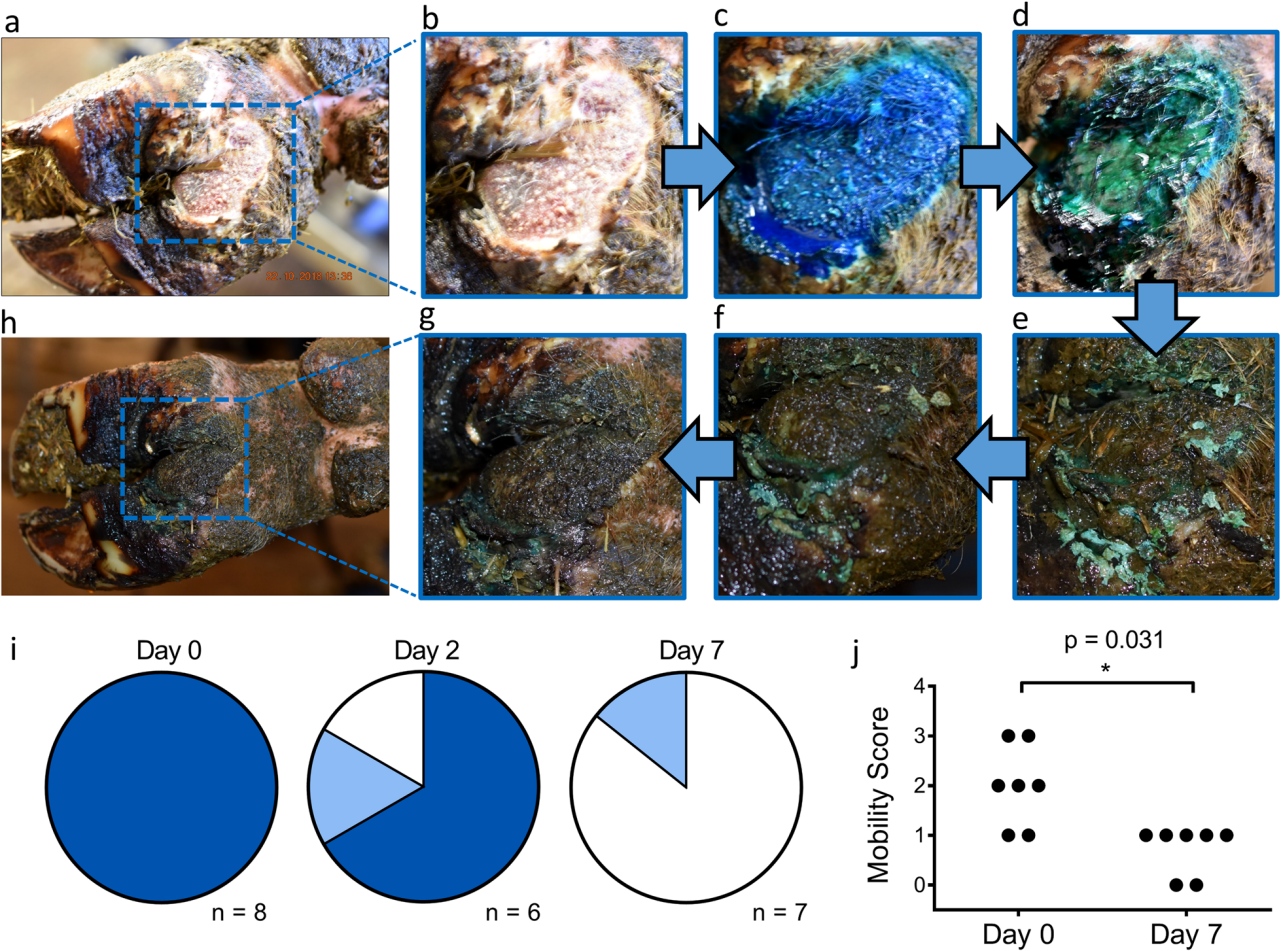
In vivo proof of principle testing in farm animals with digital dermatitis. (**a**, **b**) Digital dermatitis lesion (**c**) with antibiotic applied (**d**) immediately followed by CAZ barrier to form a bandage on day 0. The subsequent images show the exact same region (**e**) on day 2, (**f**) day 4 and (**g**, **h**) day 7. (**i**) Barrier integrity, assessed by the blind scoring of (coded) images after CAZ application (Day 0) and then at days 2 and 7. Dark blue represents effective barrier coverage; light blue is clear residual barrier and white means no obviously visible barrier. (**j**) Mobility scores on day 0 and after 7 days (n = 7), with statistical analysis by single tail Wilcoxon matched-paired test.

Finally, in vivo*,* we assessed tolerability and robustness of the CAZ barrier in 7 cows with compromised mobility (scores of one or greater—zero denoting healthy movement^26^) and active digital dermatitis lesions. Lesions were treated with chlortetracycline spray as per standard care. After 30 s, 5–10 g of CAZ barrier were applied on top of the spray. This was allowed to dry for 2 min, photographed and cows were then returned to the herd. Where possible, at days 2, 4 and 7, the lesions were gently washed and re-photographed (Fig. 3a–h). Mobility scores were recorded again at day 7. Images were graded by an independent observer as (1) effective barrier coverage, (2) residual barrier or (3) no visible barrier. In all cases CAZ adhered well upon application (Fig. 3i). By day two, 4 out of the 6 lesions that could be reviewed still had effective barrier coverage whereas, by day 7, only 1 lesion showed clear evidence of residual barrier (Fig. 3i). Throughout, there was no evidence of local adverse responses to the CAZ barrier. In fact, with the single treatment, lesions healed and for all animals at day 7, lameness was ameliorated to a significant extent (*p* = 0.03 v day 1) with mobility scores normalised to 0 or 1 (Fig. 3j).

## Discussion

Whilst rapid-set liquids, for beneficial application to lesions, are not new, the existing products (e.g. polyacrylate aerosol sprays) form weak barriers that are neither biodegradable nor antimicrobial. Moreover, they cannot be used for farm animals as they are not acceptable components of the food chain. Overall, there is an unmet need for robust, sterile liquid bandages with broad applicability across humans and other animals including, for the latter, those contributing to the human food chain.

Here, we demonstrate a novel class of antibacterial, rapid-setting, liquid bandage that utilises the natural resin, shellac, at high film former content (40% w/w) to produce durable in situ bandages when applied to synthetic surfaces, cadaver limbs and bovine wounds. Incorporating antibacterial metals (chiefly Cu^2+^ and Zn^2+^) in the formulation conveyed contact-killing properties which is also a first-in-class functionality for liquid bandages. This property is aimed at limiting the potential for continuous bacterial colonisation of the dressing, or bacterial ingress into the wound, thus aiding in the maintenance of a sterile wound environment which gives best chance to the natural healing process. It also removes the necessity for both a dressing and a bandage, as it serves these purposes in one.

Metal ion addition to the formulation also benefited physical and chemical properties of the materials by enhancing both adhesion to application sites and barrier durability in aqueous environments. We are yet to elucidate the precise mechanisms for these effects. However, it is notable that shellac’s constituent aliphatic and alicyclic acids have pKa values in the range of 5.8–6.1^27^ and that their deprotonation under neutral and alkaline conditions increase the polymer’s aqueous solubility. It is possible that in the presence of transition metals, these carboxylate moieties instead become co-ordinating bridges between neighbouring polymer strands, forming supramolecular metal organic framework (MOF)-like structures, and thereby retarding dissolution. Irrespective of the precise mechanism, this behaviour allowed us to tailor degradation rates via compositional fine-tuning (metal and shellac content) such that, in preliminary in vivo testing, our material resisted the extensive stresses of the bovine hoof environment for at least 2 days before self-degrading. Importantly, as described, it was also well tolerated, allowed wound-healing and was effective at ‘locking in’ pre-applied therapeutics. These features pave the way to much-needed one-shot combination therapy options for digital dermatitis given that the current gold standard (repetitive applications of topical antibiotic) is impractically labour intensive, whilst dressing antibiotic with traditional bandages is time consuming and runs the risk of fouling should the bandage be left in place. Self-degrading bandages obviate this risk and, as such, CAZ is a ‘first in class’ for a robust liquid bandage in several aspects.

More generally, there are many scenarios, in humans and animals, where these new materials could find usage as devices to cover wounds, lesions and burns that benefit from rapid isolation from their environment and a de facto ‘second skin’. Surgery, trauma (including burns) and disease-associated lesions (as reported here) would be major target applications. In particular, the difficult-to-get-to lesions as well as those occurring ‘in the field’, rather than a controlled clinical setting, could especially benefit from this technology. Infected and wet environments deliver a further challenge that these materials rise to especially well. Even upon exposure to an aqueous liquid before setting, the barrier formulations immediately cure and repel the fluid.

Our main clinical goal was to really challenge these new materials and to see how they would perform when applied to difficult areas, with open wounds, in an infection- and water-rich environment on a host without conscious compliance. Bovine digital dermatitis was therefore our test bed. This is a relapsing and remitting disease, and our aim, here, was proof-of-principle rather than a clinical trial but it convincingly demonstrated that the barrier is easily applied, is robust and well tolerated, locks in a pre-applied therapeutic and allows lesions to heal. Whether this will turn out to be a superior method for dealing with digital dermatitis, compared to current state-of-art management, would require a controlled trial with larger numbers but it is worth noting that Klawitter et al.^3^, cite an urgent requirement for new approaches to bandaging in this disease and we believe that the materials presented here have that potential.

## Materials and methods

### Preparation of liquid bandage formulations

All reagents were purchased from Sigma apart from ethanol (Fischer Scientific) and de-waxed (< 0.5%) shellac (A.F. Suter & Co; Shellac Dewaxed flakes HS702MB). The shellac flakes were blended to powder using a Waring Blender 8011EG.

Liquid bandages were prepared by the sequential mixing of (a) metal salts (copper, zinc and/or iron), (b) additives (e.g. triethyl citrate, ZnO), and (c) shellac, in ethanol solvent via roller mixer (Denley Spiramix 5, Thermo Scientific, UK). Soluble copper and zinc salts (acetate and chloride) were each added in quantities sufficient to produce bandage formulations at 100 mMol/kg, whilst ferric chloride was added at 66 mMol/kg (CAF and ZAF materials). Additives were employed in a number of materials with the intent of imparting additional functional benefits via (1) increased metal content: 1% ZnO, 8% triethyl citrate dispersant (CAZα), (2) presence of an organic antimicrobial and preservative: 40 mMol/kg benzoic acid—neutralised with KOH (CCβ), or (3) plasticizers: 12% polyethylene glycol (~ 400 Da), 5% glycerol (CAZρ). Finally, blended shellac was added at 40% (by weight) to disperse solutions and agitated via roller mixer for at least 24 h until homogeneity. The components of each material are tabulated in Table 1.

### Helium ion microscopy

Films were imaged using a Zeiss Orion NanoFab Helium Ion Microscope (Carl Zeiss, Cambridge, UK). The microscope was operated at an imaging voltage of 30 kV and an aperture size of 20 µm. An Everhart–Thornley (E–T) detector was used to image the samples.

### Metal release and contact killing activity against *E. coli*

The contact killing activity of antibacterial barriers was determined using an assay adapted from the international standard ASTM E2149^28^. Briefly, films were prepared by pouring 1 mL of the liquid bandage formulations in each well on a 12-well plate and allowed to dry overnight at room temperature. In parallel, a saturated *E. coli* K12 ATCC 47076 culture in Lysogeny Broth (LB), *ca*. 10^9^ CFU/mL, was prepared and diluted 100 × in LB—final concentration of 1.5 × 10^7^ CFU/mL. 1 mL of this culture was added on top of the dried shellac formulations and incubated for 24 h at 30 °C under mild agitation (80 rpm, New Brunswick Innova 4000 Incubator shaker). Bacteria concentration was determined using agar plate counting methodology, in which samples from the *E. coli* culture were decimally diluted in PBS and plated in LB agar plates for colony counting. Barriers were considered bactericidal if no bacterial colonies were detected in agar plate counting method (limit of detection = 1.7 log CFU/mL).

Samples from the bacterial culture exposed to each shellac formulation were also collected to determine copper and zinc content. These samples were diluted in 5% HNO_3_ to concentrations below 100 mg/L of the metal and quantified using inductively coupled plasma optical emission spectroscopy (Jobin Yvon Horiba Ultima 2C; Instrument SA, Longjumeau, France), employing a concentric nebulizer and cyclonic spray chamber. Plasma gas flow rate was 10 mL/min and the sample flow rate was 1 mL/min. Triplicate measurements were made for each sample and means and standard deviations calculated from these. Assay-sample concentrations were determined from matrix matched standards via their emission intensities at wavelengths of 324.754 nm (Cu) and 213.856 (Zn)^29^.

### Barrier degradation

22.5 g of material CAZ or 40% w/w shellac in ethanol were transferred to the bottom of 15- or 50-mL falcon tubes, and then spun for 1 min at 1500 rpm (Sorvall Legend RT 75006445 rotor) to ensure all material was at the bottom. The tubes were then uncapped and the formulations dried for 30 min. Next, simulated slurry (SS; 75 mMol/kg ammonium carbonate, pH 9.0 ± 0.2^30,31^ was added to obtain formulation:SS ratios (w/w) of 1/5 (12.5 ± 0.1 g), 1/10 (25.0 ± 0.1 g), 1/15 (37.5 ± 0.1 g) and 1/20 (50.0 ± 0.1 g). The tubes were then rolled for 24 h at 50 rpm (Denley Spiramix 5, Thermo Scientific, UK) at room temperature, after which they were spun for 5 min at 3500 rpm (Sorvall Legend RT 75006445 rotor). The supernatant was disposed of and the remaining formulation was dried at 45 °C till constant weight.

Relative mass losses were determined against CAZ or 40% w/w shellac (2.5 g) solutions that were also dried to constant weight in identical tubes but not exposed to SS.

### Antibiotic lock-in

Bovine cadaver limbs were employed in ex vivo antibiotic lock-in experiments. An antibiotic spray formulation used for treating digital dermatitis (Animedazon Spray, containing Patent Blue V dye and chlortetracycline active) was applied to limbs by spraying, for 2 s, a region commonly afflicted with lesions (the area between the dew claws and the interdigital cleft). Spray-can masses were recorded before and after applications to control for the amount of antibiotic applied. Upon spraying, the antibiotic formulation was allowed to dry for 60 s and then limbs were either: (a) Control Group (n = 4): directly immersed in 1 L of simulated slurry (SS; 75 mMol/kg ammonium carbonate) or (b) Barrier Group (n = 4): treated with a barrier formulation (15 ± 5 g of CAZ) applied to cover the antibiotic spray-site, allowed to dry for a further 120 s and then immersed in 1 L of SS. After 1 h of immersion, 10 mL aliquots of SS were taken for each limb and the assay stopped. Samples were analysed for absorbance to determine the proportion of antibiotic formulation lost to the simulated slurry fluid; the strong and characteristic spectrochemical profile of Patent Blue V (λ max_H2O_ = 639 nm^32^) made absorbance measurements a convenient proxy for antibiotic losses. Absorbance standards were prepared by serial dilution of weighed masses of antibiotic spray in the simulated slurry solution. Subsequently, sextuplicate aliquots (200 μL each) from each standard and assay sample were plated (Corning Co-star 96 well) and measured for absorbance between 350 and 850 nm. Raw spectra had noisy and variable baselines (due to organic contaminants on the limbs) and required processing in MATLAB (Savitsky Golay filtering followed by a baseline subtraction with 2nd order polynomials) to obtain reliable absorbance maxima. Finally, linear standard curves (concentration vs absorbance at 639 nm) were used to generate loss data for assay samples. The datasets comprised limited observations so, to avoid making assumptions regarding their distributions, statistical analyses were performed using a non-parametric method—namely two-tailed Mann–Whitney *U* tests. A value of *P* < 0.05 was considered statistically significant. All statistical analyses were performed using Prism 6 (GraphPad Prism Software).

### In vivo farm study

Eight dairy cows suffering from digital dermatitis and with impaired mobility were randomly selected and isolated for treatment at the Cambridge University Farm. All work was carried out under ethics approval from the Ethics & Welfare Committee from Department of Veterinary Medicine at the University of Cambridge (CR302—‘Digital dermatitis treatment using liquid barrier and copper/zinc actives in cattle’) and adhering to AAALAC standards^33^. Briefly, for each animal, limbs were immobilised using a standard foot trimming crush and water-hosed to remove slurry and other detritus. Animedazon Spray, containing 2.45% w/w chlortetracycline, was applied and allowed to dry for a few seconds as per standard veterinary care. Subsequently, 5–10 g of CAZ was applied – such that lesions were fully covered. After one minute, cows were released and allowed back into the yard. Photographs were taken during this process and during the follow up at days 2, and 7, and subsequently used for single blind assessment of barrier integrity on a three point scale of: (A) effective barrier coverage, (B) clear residual barrier or (C) no obviously visible barrier. In addition, mobility scores for the animals were obtained from veterinarians at day 0 (before application) and day 7. Animal mobility was assessed by veterinarians using a standard scoring system^34^ described in Table 2, in which scores varied from 0 (good mobility) to 3 (severely impaired mobility). Mobility score data (day 0 vs day 7) was discrete and comprised limited observations so could not be assumed to follow a normal distribution, thus statistical analysis was performed using Wilcoxon matched-pairs signed-rank test. Since intervention (including conventional antibiotic treatment) would not result in clinical worsening, single-tail testing was employed to maximise power from the small sample size. A value of *P* < 0.05 was considered statistically significant. As previously, statistical analyses were performed using Prism 6 (GraphPad Prism Software).

**Table 2:** Description of the mobility scoring system used in this study^34^.

Score	Mobility category	Description
0	Good mobility	Walks with even weight bearing and rhythm on all four feet, with a flat back. Long, fluid strides possible
1	Imperfect mobility	Steps uneven (rhythm or weight bearing) or strides shortened; affected limb or limbs not immediately identifiable
2	Impaired mobility	Uneven weight bearing on a limb that is immediately identifiable and or/obviously shortened strides (usually with an arch to the centre of the back)
3	Severely impaired mobility	Unable to walk as fast as a brisk human pace (cannot keep up with the healthy herd) and signs of score 2

## Supplementary information

Supplementary Information.
