# Supplementary material for: Robust rapid-setting antibacterial liquid bandages
Source: Sci Rep. 2020 Sep 15;10:15067. doi: 10.1038/s41598-020-71586-7 (PMC7492242; doi:10.1038/s41598-020-71586-7)
Supplement: Supplementary file 1 — Supplementary Information. [file 41598_2020_71586_MOESM1_ESM.docx]

# Robust Rapid-Setting Antibacterial Liquid Bandages

Carlos A. P. Bastos^1+^*, William D. Thom ^1+^, Beth Reilly ^1^, Iris L. Batalha ^2^, Maedee L. Burge Rogers ^1^, Ian S. McCrone ^1^, Nuno Faria^1¥^ and Jonathan J. Powell^1^*^¥^

^1^ Department of Veterinary Medicine, University of Cambridge, Madingley Road, Cambridge CB3 0ES, UK.

^2^ Nanoscience Centre, Department of Engineering, 11 J. J. Thomson Avenue, Cambridge CB3 0FF, UK.

^¥^ or ^+^ denote equal authorship.

* Lead and corresponding authors:

Carlos A. P. Bastos: [capb2@cam.ac.uk](mailto:capb2@cam.ac.uk)

Jonathan J Powell: [jjp37@cam.ac.uk](mailto:jjp37@cam.ac.uk)

Phone: +44 1223760003

**Supplementary Information**

**Supplementary Information 1 – Formulation retention upon application**

Barrier retention in a bitumen surface was tested at a slope angle of 63°. Our lead material CAZ was compared to materials containing shellac only (no metals) at equivalent concentrations 40% and 50%. 3g of barrier were poured on the carpet and the material losses by mass (non-retained in the surface) was measured. This work demonstrated that by adding the metals, the amount of material retained was higher than at equivalent (40%) or higher (50%) shellac concentrations with no metals (Figure S1).

**Figure S1.** Example of mass losses upon application of 3 g of barrier formulation to a 30 cm^2^ bitumen surface oriented at a slope angle of 63°. CAZ formulation is composed of copper acetate and zinc chloride in 40% (w/w) Shellac, as described in Materials and Methods section and Table 1. 40% and 50% ethanol shellac solutions were prepared without metal ion addition.

**Supplementary Information 2 – Lesion information form *in vivo* study**

Animals were randomly selected based on visible movement impairment. Lesions were assessed by a veterinarian clinician and information from initial infection was not available.

**Table S1** – Information about digital dermatitis lesions for each animal.

| Animal Number | Leg | Lesion Grade |
| --- | --- | --- |
| 1 | Back left | M2 |
| 2 | Back right | M4 |
| 3 | Back right | M2 |
| 4 | Back left | M4 |
| 5 | Back right | M2-3 |
| 6 | Back left | M4 |
| 7 | Back right | M2 |
| 7 | Back left | M3 |
